# Hierarchical Modelling of COVID-19 Death Risk in India in the Early Phase of the Pandemic

**DOI:** 10.1057/s41287-020-00333-5

**Published:** 2020-12-15

**Authors:** Wendy Olsen, Manasi Bera, Amaresh Dubey, Jihye Kim, Arkadiusz Wiśniowski, Purva Yadav

**Affiliations:** 1 Department of Social Statistics, University of Manchester, Manchester, M13 9PL UK; 2 Indian Institute of Dalit Studies, D-II/1, Road No-4, Andrews Ganj, New Delhi, 110049 India; 3 Centre for the Study of Regional Development, School of Social Sciences, Jawaharlal Nehru University, Delhi, India

**Keywords:** Epidemic modelling, Latent variable, Hierarchical model, Bayesian model, Data combining, Severe COVID-19, SARS-CoV2 virus pandemic, India, Pandemic

## Abstract

We improve upon the modelling of India’s pandemic vulnerability. Our model is multidisciplinary and recognises the nested levels of the epidemic. We create a model of the risk of severe COVID-19 and death, instead of a model of transmission. Our model allows for socio-demographic-group differentials in risk, obesity and underweight people, morbidity status and other conditioning regional and lifestyle factors. We build a hierarchical multilevel model of severe COVID-19 cases, using three different data sources: the National Family Health Survey for 2015/16, Census data for 2011 and data for COVID-19 deaths obtained cumulatively until June 2020. We provide results for 11 states of India, enabling best-yet targeting of policy actions. COVID-19 deaths in north and central India were higher in areas with older and overweight populations, and were more common among people with pre-existing health conditions, or who smoke, or who live in urban areas. Policy experts may both want to ‘follow World Health Organisation advice’ and yet also use disaggregated and spatially specific data to improve wellbeing outcomes during the pandemic. The future uses of our innovative data-combining model are numerous.

**Electronic supplementary material:**

The online version of this article (10.1057/s41287-020-00333-5) contains supplementary material, which is available to authorized users.

## Resumé

Dans le contexte du développement international, on peut améliorer la modélisation de la vulnérabilité à une pandémie en combinant différentes disciplines, en combinant des données et en reconnaissant les nombreux niveaux imbriqués de l'épidémie. Des modèles de transmission ont été élaborés à l’échelle nationale ou pour plusieurs pays. A l’inverse, nous construisons un modèle permettant de prendre en compte les différents niveaux de risque selon les groupes sociaux, ainsi que le conditionnement des facteurs régionaux et des facteurs liés au mode de vie. La forme grave de la COVID-19 est notre résultat clé innovant. Nous utilisons trois sources de données simultanément: l’enquête nationale sur la santé des familles en Inde, le recensement de la population indienne de 2011 et les décès liés à l’épidémie de COVID-19. Nous fournissons des résultats pour 11 États en Inde, ce qui permet un meilleur ciblage des actions politiques. Les utilisations futures de ces modèles sont nombreuses. Dans le nord et le centre de l'Inde, les décès liés à la COVID-19 étaient plus nombreux dans les régions avec populations âgées et populations en surpoids. Ces décès étaient plus fréquents chez les personnes ayant déjà des problèmes de santé, ou chez celles qui fument ou qui vivent dans les zones urbaines. Les experts en politiques publiques pourront souhaiter à la fois « suivre les conseils de l'Organisation mondiale de la santé » tout en utilisant des données désagrégées et spatiales pour améliorer les résultats en matière de bien-être pendant la pandémie.

## Introduction

In India, all persons experiencing symptoms of COVID-19 are required to attend at government-managed COVID-19 testing sites. The state governments have the task of managing COVID-19 evidence, including individual cases of infection, and they record the COVID-19-related deaths. Then the national Ministry of Health and Family Welfare handles the collation of state evidence. It releases both summary data and public-health advice via webpages. Individual states also release data on COVID-19 cases in the respective states. However, the data provided publicly are often not disaggregated by sex, age and districts. Similarly, many migrant workers who have been living in urban areas separate from their household of origin and moved back to village during the pandemic were generally not tracked, although temporary migrant registration lists were kept for some states. There were political demands to manage the transportation of migrants on key routes. Data summaries were provided at District level via third-party organisations not benchmarked by government (e.g. How India Lives 2020). Data thus form a key part of the machinations of managing a pandemic. In this paper, we suggest how a mixture of the data from different sources can be analysed: administrative data, random-sample microdata and the actual COVID-19 deaths data. Data for deaths in India are modelled by using a hierarchical model within an information-optimising Bayesian inferential framework. Such models combining aggregate and individual-level data are rare in the COVID-19 literature. Our model can open up a wide range of variant models.

The paper begins with a review of selected modelling options including epidemic models and social patterns of health and social care in epidemics, and discusses the framework used for the study. Next, the data and methods are introduced for our analysis of 11 Indian states, based on data from June 17, 2020. The research question for this section is what social factors contribute to the higher rates of COVID-19 deaths, both directly at the time of this disease and also through underlying long-term processes of exposure to health risks and lifestyle factors that created vulnerability. Using a Bayesian hierarchical model, we develop a latent vulnerability index linked to district death counts. Such an index cannot be constructed using more traditional modelling methods. The results are discussed in detail. Next, we discuss policy implications both at the India level and cross-nationally. The paper closes with reflections on the broad purpose of development research in the COVID-19 area.

The epidemic progressed differently in different countries. In India, the progress of COVID-19 spread has been different in various regions. The case counts rose from 137,000 to 307,000, and deaths rose from 4,800 to 9,900 within the four weeks from 20 May to 17 June 2020 (How India Lives 2020) with a large proportion of the infections reported from the North, Northwest and Central regions. India’s deaths grew to 23,700 by 14 July 2020 (time of writing), creating a national cumulative death rate of 1.7 deaths per 100,000 population on that date.^1^

**Fig. 1:**
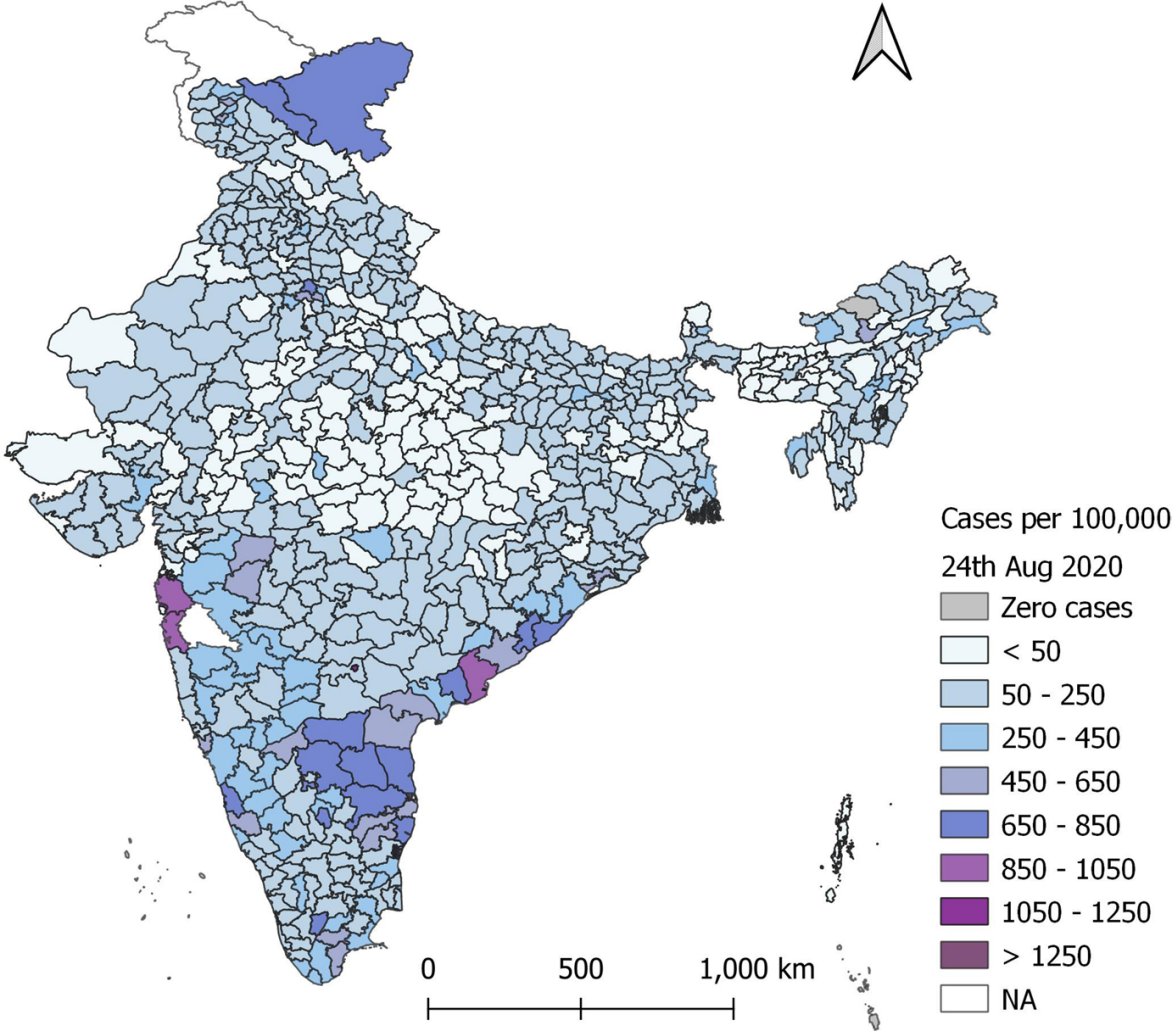
Cumulative Indian COVID-19 cases per 100,000 population, by districts (August 2020)

In India, the overall case rates—cases per 100,000 people—grew rapidly. Figure 1 illustrates the spatial spread of cases using the latest data (August 2020), while the bulk of this paper is based on June 2020 cross-sectional data. The model presented below is based on India’s deaths data. Our strategic choice to model deaths arose from difficulties with modelling transmission and also arose due to problems with the data on cases of COVID-19, discussed in detail in the paper.

As a policy measure, the Indian lockdown started at a less than 24 h notice on 25 March 2020 and ran until 30 June 2020 with the unlocking phased in from 1 June (GoI 2020a). All travel in public was barred, except essential and approved travel. Schools and other academic institutions, restaurants and bars were closed. Government offices were also closed, excepting the critical ones dealing with health, which functioned with skeletal staff. Migrants who tried to return to their original homes were actively discouraged, and for some time migrants were stopped from using public transport because of the travel ban. Persons above age 65 or below age 10, persons with comorbidities and those otherwise vulnerable were advised to stay at home (GoI 2020b). COVID-19 helpline numbers were provided at national, state and district levels. A contract-tracing app was made mandatory, providing information and an emergency response (GoI 2020c).

### Models of Transmission and Severity of SARS-CoV-19

Three kinds of mathematical models—the COVID-19 impact model, the SEIR model and the SEIR model combined with agent-based modelling—were used to study the rates of transmission of the virus. All three types of models use both the infections and deaths as stages in the disease process. The models include variables on ‘cases of COVID-19’ and ‘deaths from COVID-19’, suggesting an ultimate focus on human wellbeing. The ratio of these two variables is the case fatality ratio (CFR, i.e. deaths per person infected, e.g. Verity et al. 2020). The *Lancet* editorial of 24/1/2020 (Wang et al. 2020) noted a CFR of 2.9% in the Wuhan epidemic up to then. It stated that for the first 41 cases the rate was 15%. The CFR reported for the UK has been 14%, while India’s has been 2.6% (Roser et al. 2020, 14/07/2020). The underlying data for the national CFRs of both UK and India may be unreliable. A particular problem arises if the testing system does not allow some categories of people to get tested promptly. In the case of India, it is likely that the deaths data are more inclusive or more accurate than the data on positive COVID-19 tests. In many countries like India, the exposure (cases) have not been precisely estimated. By contrast, Russell et al. (2020) provided the accurate case fatality ratios for the Diamond Princess cruise ship, where there was eventually 100% testing after a severe outbreak in a bounded group of people. The CFR and cumulative cases per capita play a key role as parameters in models. We can also model the *impact* of COVID-19 based on models of the spread of the disease (Pellis et al. 2020). Our hierarchical model of COVID-19-related deaths in this paper is indeed a model of the impact of COVID-19 on diverse groups of people. It is not a model of transmission. However, we first review the literature which includes the transmission stage as well as the stages of severe disease and death or recovery; then we introduce our model.

The SEIR model of the spread of disease focuses on susceptibility, exposure, the infection stage, and death or recovery (the four SEIR compartments, Brauer et al. 2008). The infection rate can be analysed or forecast by measuring the rate or reduction of contagion via distancing, hand-washing, lockdown and other policy tools. These are called the non-pharmaceutical interventions. Hill’s online app (2020) and the apps for forecasting by Pueyo (2020) and Neher (2020a; b) enable countrywise comparisons of these stages. The SEIR model adapts a traditional epidemiological model to allow for social parameters. The equations for the model use time-series data (Wu et al. 2020, pp. 247–248; Hill 2020).

The count of each compartment’s people is based on a proportion of the previous compartment, depending on who has been exposed: children, adults, old people; and their body types, home types, past illnesses and other factors. The basic reproductive number, R0, summarises how far each infected case spreads the virus to further cases (Wu et al. 2020). The model’s summation of exposure rates allows disaggregating each overall parameter (Pellis et al. 2020).

Data from the Diamond Princess cruise ship were widely used to demonstrate that children rarely tested positive for COVID-19 (Russell et al. 2020). The contagion was biased towards the oldest age groups. There was little sex bias. These data were considered definitive. However, deaths were even more strongly biased towards older people, and in many countries towards men. The risk of death is impacted not only by the social and biological contagion process but also by the experience of illness and health treatments, and the body’s response. Social factors enter into the modelling of the severity of the illness.

The SEIR model has had a lot of close attention (see Alimentarius 2012; Amirian 2020; WHO 2008, 2020a, b). Currently, experts disagree on whether this virus is contagious or not during its latent period. As a result, public-health policies on non-pharmaceutical interventions have been questioned because how this virus spreads is still fundamentally unclear.

Hellewell et al. (2020) concluded that isolation and contact-tracing would not succeed in limiting the virus spread, if the R0 number was too high. They assumed R0 was from 1.5 to 3.5, based on the experience in Wuhan, China. For countries like India, we should model the effects of a lockdown, or else the mathematical model of transmission could be too much of a black-box style model (facts-in-shape-facts-out). Then, if one inputs false claims (which we call ficts) then one gets ficts out (Olsen and Morgan 2005). Nevertheless, models help with thinking through interrelationships, exposing correlations and creating an openness to new parameters that policy may focus upon. Being exposed to the virus is a vital and necessary step towards the outcome of death from the virus. So, the models of transmission and disease severity are intrinsically linked.

The lack of clarity about the role of asymptomatic cases of COVID-19, or the length of the latent period of the virus, tended to make the value of a transmission model for India’s epidemic questionable. We instead focused on making a model of the tendency to have a death reported to be due to COVID-19. The background factors encompass those factors which increase, or reduce, transmission. Now, we discuss some of these factors.

In India, there is concern that living in joint families would lead to high rates of transmission (Singh and Adhikari 2020). Hill et al. (2010) also showed decisively that social networks matter very much to the way that a virus is passed on. Pujari and Shekatkar (2020) analysed social network patterns affecting viral infections in India. Verity et al. (2020, p. 3) said that the cruise ship Diamond Princess data gave the best insight to the biological processes of exposure and infection. There, the 100% testing enabled asymptomatic cases to be included in the case rate data. There is a nomothetic assumption here, which means that all persons are alike, no matter which country. They assumed that biological data from Diamond Princess are valid everywhere. The latent period length was measured in the Diamond Princess data, and this became a key variable in the online forecasting models. This parameter could, in turn, be potentially differentiated by social group, body type, genetic features and immune response. The conceptual framing can be opened up to local, social and lifestyle differentiation. In our view, a multidisciplinary model would tend to obviate the usefulness of the original nomothetic or biological assumptions. The reality is that the model for India should extend beyond the biological variables.

The normative overlay on most of the above models is that recovery is good, while the interpretation of the rest of the underlying mechanisms is complex. One may even want to get the disease and fight it off so that one might become immune. This complex normative possibility raised much discussion: would it be better to expose more than  50% of the population so that they could become immune? Or would a country be better advised to use non-pharmaceutical interventions, such as lockdown and distancing, until a vaccine could be found? The decision rests in part upon the disease symptoms and the severity of impacts. The costs of each case are relevant. Decisions rest in part on the future vision of the possibility of finding a vaccine and being able to produce or buy it. Most analysts try to reduce the infection rates in order to lessen the burden on the healthcare systems, as well as improve wellbeing and decrease the number of potentially lost lives arising from vulnerable people getting infected. The neoliberal angle on these ethical issues is discussed further in Olsen (2020). See also Chang and Grabel (2014), who contrast neoliberal policies, which in current situation are perhaps more likely to assume that a vaccine is available relatively soon, with alternatives. Thus, the undertone of the expanded SEIR model is that the norms are complex and an informed, evidence-based discussion is needed (Olsen 2007).

An SEIR variant model by Tomas Pueyo (2020) became popular*.* Pueyo (2020) stressed that lockdown lowers the daily rise in cases, but afterwards, the epidemic will re-surge (*ibid.*). The model had cycles of rising and declining case rates. Hill (2020) and Dropkin (2020) focused on a single exponential growth stage. A key problem is that exponential growth is the pattern to which the virus transmission reverts if mitigation measures are relaxed.

Aswi et al. (2020) showed that the correlated geographically contiguous spread of a different disease, Dengue Fever, meant that a spatial autoregression model would help in forecasting a virus spread pattern. They innovatively used a Markov Chain Monte Carlo (MCMC) algorithm for a Bayesian estimation to make large models tractable without requiring a single maximum-likelihood function to encapsulate all the equations at once. Rajendrakumar et al. (2020) also illustrate hierarchical Bayesian models.

Usefully, there is a third model type. It uses agent-based modelling to make a forward trajectory for the parameters corresponding to each social group. For example, whether schools are open or closed will influence the R0 number of the families in a specific neighbourhood in each time-period. Agent-based models have sub-group differences within the population and they allow step-wise change over time in key parameters. This type of model can blend the disaggregated findings of agent-simulation with the SEIR analysis over time (Ferguson et al. 2020; Flaxman et al. 2020). Baseline characteristics from northern Italy were used to set some of the parameters (Grasselli et al. 2020). Each person’s background factors, such as diabetes, lung problems or tuberculosis, were ignored in this model (c.f. Liu et al. 2020), where the focus was on infection rates, not severity. By contrast, our model lacks agent-based forecasts and is focused on the severe forms of COVID-19.

Ferguson et al. (2020) showed that after an initial improvement there would be a second wave of viral infections. Ferguson’s model was superior in noting social types, social-networking patterns and social groups (Ferguson et al. 2020, p. 4; Adam 2020). Adaptations were also made to allow for people being more or less contiguous at each point in time. It is a model based partly on contagion. However, it was weak in its coverage of the lockdown impact.

With so many parameters, any of the above models could be tweaked, leading to a lower or higher overall case fatality rate. Criticisms arose because there was a worry that subjective or political factors were entering into scientific modelling. Extrapolations for India based purely on China data would be troubling because social-networking patterns in India might be different. Therefore, the transmission rates would be different (Kretzschmar et al. 2020; Singh and Adhikari 2020). The model in the present paper focuses on severity. It emphasises the advantage of using information on the COVID-19 death rate and the age structure along with other population characteristics such as health, lifestyle, urban location and social structure, that are unique to a given population. These characteristics allow a better understanding of disease transmission and severity.

Our review of literature showed a paucity of research on the severity of the COVID-19 disease. Social forms of marginalisation of groups of people can lead to severe health risks due to some having a weak economic position, poor living and working conditions, and poor psychological and physical health outcomes in the years preceding the COVID-19 epidemic. The National Family Health Survey-4 (NFHS 2015/16) found the highest malnutrition, mortality, low level of obstetric care and poor healthcare services utilisation among the Scheduled Tribes (ST) and Scheduled Castes (SC) groups (IIPS & ICF 2017; Acharya 2018). Social groups who face social exclusion in India also have had historical, entrenched inequality of incomes (Thorat and Dubey 2012). They have been the subject of concerted policies which have decreased inequality of household incomes by reserving jobs for people (mainly men) of the SC and ST groups while also progressively enhancing the social separation of the identified groups (Booroah et al. 2007).

Further, in addition to customary practices, dearth of healthcare and transportation facilities help to explain the high prevalence of untreated chronic morbidity among ST people (Raushan and Acharya 2019). Low age of death and poorer health outcomes of elderly among these groups (Borooah 2010) indicate higher vulnerability. Exposure to COVID-19 infection may add to the existing health risks for the marginalised groups. Also, these groups are more likely to be directly exposed to the virus due to their engagement in cleaning and sanitation work.

### Discussion of Models of Transmission and Severity

Models help to organise the thoughts of scientists and experts who are crossing disciplinary boundaries. They expose and make measurable key elements in social and socio-biological processes. Each theory is general, in that it can only handle a certain limited amount of detail. Some efforts to make models super-complex have succeeded in the sense of being socially and politically useful. Other efforts to achieve complexity have failed due to ignoring the existing literature on complexity. The specific additions to knowledge of complexity theory include: First, each process can be broken down into a series of stages, and progression is path-dependent. Second, one can pay attention to different types of units at one time point, which can lead to a wide set of trajectories; this is a disaggregated, multilevel form of path-dependence. As a result, qualitative factors can be incorporated in the models, and trajectory-switching can be tuned to respond to recorded multinomial and modal measures. Third, latent measures can be used.

**Fig. 2:**
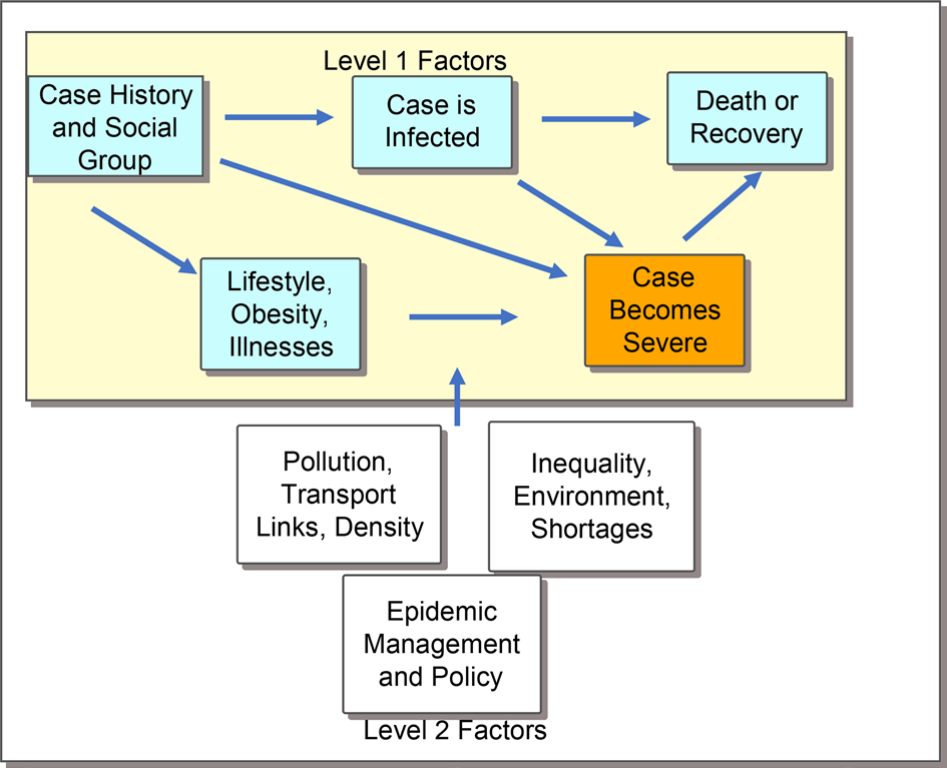
A hierarchical model of COVID-19 contagion and severity. *Notes* Level 1 is persons, and Level 2 is districts. The hierarchical (multilevel) model can be estimated cross-sectionally or over time. Our models 1–2 use a subset of the above variables cross-sectionally. Arrows represent influence over time; the orange variable is latent and we estimate the risk of death, see Eqs. 1–3

Figure 2 illustrates the concept of mediated effects upon COVID-19 disease severity. The mediation occurs when the impact of a variable (e.g. smoking) is both direct and indirect. Moderated effects occur if a local condition, such as pollution, increases the pre-existing diseases and makes people more susceptible.

In Fig. 2, we present a general conceptual framework for a multilevel model. It has district factors (Level 2) in the lower frame, and individual factors (Level 1) along with social-group histories in the upper frame. In this model, obesity may reflect either lifestyles or a long-term accumulation of genetic features, or both. The average obesity rate in a district reflects wider patterns of social inequality. Among the district-level factors, Fig. 2 names several, but we operationalised only four of these in this paper. These were the district-level migration, the urban percentage, the obese percentage and the percentage underweight according to the WHO standards. This paper offers a proof of concept for the Bayesian combined data models and the proposed multilevel approach can be extended in multiple ways.

The variable in orange is ‘severe COVID-19’ status, and its risk is reflected in a latent variable. Death counts are used as evidence about the incidence of severe COVID-19. Such models with two or more dependent variables are broadly classed as structural equation models. The equations (below) show exactly how the latent variable and the counts are integrated along with the other empirical data. Bayesian modelling examples for counts of a dependent variable are found in Bryant and Zhang (2018). Illustrations of Bayesian estimation with multilevel models are found in Gelman et al. (2013), while Wiśniowski et al. (2016) provide an example of combining multiple data sources.

In development theory and practice, we approach interpreting such a model using complexity theory. It is usual in complexity theory to be aware of nested systems, multiple levels, and the upward and downward effects of each ‘layer’ or type of entity, e.g. human bodies, hospitals, state policies and international travel quarantines all affect each other.

Complexity theory also tells us that there is a difference between ‘unobserved’ and ‘unobservable’ features of the world. We can have algebraic representations of ‘unobservable’ features while making models correspond to observed factors at the same time. The butterfly effect and path dependence become second nature to complexity theorists. Much simpler algebraic models, such as extrapolations over time, are best-guess estimates of complexly overlapping, interacting systems.

Models of SARS-CoV2 and COVID-19 impact have been a way of engaging in dialogue with public-health experts who were determined to avoid letting the exponential growth process repeat itself. Many experts want to reduce human suffering. In human capabilities theory, we stress the improvement of human lives, which can have a global scope, as well as appreciate that locally situated standpoints also underpin knowledge (Olsen 2019; Nussbaum 1999). In capabilities theory, respect for all agents means a role can be played by translating models from scientific expert to lay language. There is a key role for asking to obtain new, better data. Capabilities theory has been adopted by the Sustainable Development Goals campaign (SDGs) and has supplanted the older Gross Domestic Product (GDP) focus in development theory and practice. The SDGs, in turn, have created a sense of awareness of diverse voices in development. Capabilities theory is consistent with the remit of development journals as it applies transdisciplinarity. Epidemiology, public health, managing critical care and general practitioner services are all aimed at reducing suffering and improving capabilities. They similarly share the aim of promoting health from the start of life.

The best-yet knowledge rooted in modelling exercises can be presented in tabular, mapping and graphical formats. Our illustration is based on the Indian case. In the next sections, we attempt to disaggregate one impact of COVID-19 using secondary sources. Because India is a large country, we carry out the modelling exercise on one block of 11 states. Such models can, in principle, be estimated for large and small geographic areas. The model is highly innovative because it rests upon combining data from various sources by using novel methods, as well as the above-listed theories.

### Data

The model links the district-level counts of COVID-19 deaths (without any further characteristics) in India to the individuals’ physical, demographic, and socio-economic status and other risk factors at the district level. The primary dataset of this study is the National Family Health Survey (NFHS)-4 2015/16 that provides individual-level variables such as family wealth, adult health, child health and reproductive health. The NFHS in India has been fielded since 1992/93, and we use the one of 2015–2016. It has a large sample size: the NFHS-4 surveyed women aged 15–49 (*N* = 699,686) and men aged 15–54 (*N* = 122,122), and household members (household *N* = 601,059, household members *N* = 2,869,043, see Table A.1 in Online Annex, available at https://github.com/a-wis/Covid19-India). The NFHS-4 provides a couples’ dataset, in which the total number of individual cases is 127,300 for all states. Among them, we choose 11 states spread across the Northern, Central, Western and part of Eastern regions—Bihar, Chhattisgarh, Delhi, Gujarat, Haryana, Jharkhand, Madhya Pradesh, Maharashtra, Punjab, Rajasthan and Uttar Pradesh. The sample size is 70,980 individuals in 11 states. Among them, 1,143 cases lacking body-mass index (BMI) information are excluded. Thus, the final *N* is 69,837 (males: 34,715, females: 35,122).

Also, we use the 2011 Census data to obtain the proportion aged 65 years and above in each district. As we regress the current death data as of June 17, 2020, the 2011 Census data on the age distribution and population size should be adjusted for 2020. To project the 2020 population, we calculate the monthly growth rate of the population in each district between the last two censuses of 2001 and 2011, applying that to reach a 2020 population. For the new districts formed between 2001 and 2011, the population of new districts (with a common origin district) is added first, and its growth (2011–2020) over the origin district population is then calculated.

Individual-level variables include sex, urban residency, social group—scheduled caste (SC), or scheduled tribes (ST), smoking, health indicators, obesity, underweight and economic poverty as indicated by being in the lowest category of wealth index. A short explanation of the two social-group variables SC and ST is needed. Indian society evolved historically around a social hierarchy that in modern times became interpreted as castes. The most important features of the caste system in India has been that a large proportion of the population at the supposedly “lowest” ladder of the caste hierarchy suffered social stigma, two dimensions of which were untouchability and persistent economic and political exclusion. In turn, “untouchability” arose as a social feature of certain belief systems in which one group was considered impure by another group of people. Physical contact was avoided. Practicing untouchability was declared unconstitutional in 1955. The Indian Constitution included a list of specific caste names, and these ‘scheduled’ castes were to be denoted as Scheduled Caste (SC) people. The SC people then lived for some decades with social stigma and apparent or perceived backwardness, as well as entrenched and continuing inequality. In the literature and in politics, the SC people are also referred to as *Dalits*. They account for just under 20% of the Indian population. In addition to the Scheduled Castes (SCs) or Dalits, another group known as the Schedule Tribes has been considered by some as “outside” the main Indian social hierarchy. These people, also called Adivasis, were often considered or perceived to be somehow backward or remote, and mostly were thought to inhabit more isolated forest areas of India. They were designated as Scheduled Tribes in the Indian Constitution. They currently account for around 8% of population. The SC and ST people are numerous among those who migrate to find work within India, the phenomenon known as circular migration.

Our variables that reflect the urban residence or the SC or ST identity of a person are estimated at an individual level. The urban population is almost 30% of the total population in the 11 States. Dalit and Adivasi people form 19% and 14% of the total population in that area, respectively. We include several health-related variables which do not have correlations between them (see Table A.2 in Online Annex). These health indicators were shown in international COVID-19 literature to presage higher risk of death from the new disease.

Smoking, a binary variable, indicates whether people smoke any of tobacco, cigarettes, pipe, chewing tobacco or snuff, or not. Obesity indicates whether the body-mass index (BMI) is 30 or more, or not. If people’s BMI is under 18, they are underweight. The BMI thresholds are set by the WHO at the international level.^2^ We created an index of pre-existing health conditions using confirmatory factor analysis. Manifest variables were five key diseases—blood pressure, diabetes, asthma, heart disease and cancer. Next, quintile groups were assigned categorical indicators 0 to 4 on this scale, higher scores meaning more chronic disease. Similarly, the wealth index provided in the NFHS 2015/16 is calculated by a principal component analysis, basing the score on numerous consumer goods including vehicles along with housing characteristics (drinking water, toilet and flooring; IIPS & ICF 2017, p. 16). Our dummy variable indicates people belonging to the lowest wealth quintile.

District-level variables include obesity, underweight, the proportion of the aged population (ages 65+) and the percentage of migrants. Here, obesity and underweight are based on district-level average prevalence. They are obtained from the NFHS household members’ dataset which has a large samples size. The variable obesity means the proportion of people who have a BMI of 30 or more; underweight is the proportion of people who have a BMI of less than 18. We also estimated separate models using individual obesity and underweight from NFHS data (Online Annex, Table A.5.), and we compared the results with using those at the district level. The proportion of the population aged 65 or above is obtained from the Indian Census, in consideration of population growth between 2011 and 2020. Also, the Census 2011 provides the proportion of in-migrants in each district. In Indian Census 2011, each person was asked if they have moved in from elsewhere. The period considered here is 0–9 years recall up to 2011. Migration is a binary, for each person, and the aggregate number of in-migrants at district level is recorded in Census tables. We do not use the rural and urban differentiated migrant data.

Table 1 shows the means of key variables, including counts of deaths. Online Annex Table A.2. shows the correlation matrix.

**Table 1:** Summary of the variables in the regression model

Variable	Mean	SD	Min	Max	*N*	Description
Deaths	24.22	189.14	0	3,166	345	Counts of deaths due to COVID-19 in the district (median = 1)
Female	0.50	0.50	0	1	69,837	Male = 0, female = 1
Urban	0.29	0.46	0	1	69,837	Person’s residence is rural = 0, urban = 1
SC (Dalits)	0.19	0.40	0	1	69,837	Other groups = 0, scheduled caste (SC or Dalit) = 1
ST (Adivasi people)	0.14	0.34	0	1	69,837	Other groups = 0, scheduled tribes (ST or Adivasi) = 1
Smoking	0.35	0.48	0	1	69,837	Non-smoker = 0, smoker = 1
Ill-health	0.42	1.22	0	4	69,837	Confirmatory factor index of blood pressure, diabetes, asthma, heart disease, cancer (0–4)
Low assets	0.23	0.42	0	1	69,837	The lowest quintile of wealth is coded as 1, and the four higher asset quintiles = 0
Obesity	0.04	0.19	0	1	69,837	BMI ≥ 30 at individual level = 1, others = 0
Underweight	0.14	0.35	0	1	69,837	BMI < 18 at individual level = 1, others = 0
Age 65+	0.05	0.01	0.02	0.12	345	The proportion of population of ages 65 + living in the district
Migration	0.09	0.04	0.02	0.32	345	The proportion of in-migrants (0–9 years) living in the district
Obesity%	0.04	0.03	0	0.14	345	The proportion of people living in the district with BMI ≥ 30
Underweight%	0.20	0.06	0.05	0.37	345	The proportion of people living in the district with BMI < 18

*Sources* (1) NFHS 2015/16 couples’ individual data; (2) deaths—howindialives.com (accessed on the 17th of June); (3) age 65+ and migration—Indian Census 2011, summaries at District level

*Notes* The reference category is coded as “0” in all cases

## Methods

The models were constructed using confirmatory methods, i.e. as well-theorised attempts to mimic the real situation. We planned to combine actual measurements of COVID-19-related counts of cumulative deaths (by district within state) and individual COVID-19 case counts with the latest NFHS data. The dependent variable here is the risk of death from COVID-19 and the independent variables consist of a set of individual-level and district-level factors affecting the death risk. We assumed that by including age-group data and a rural vs urban indicator at the individual level, we would be adjusting for the age structure of the more and less urbanised districts. Further hypotheses based on the medical and epidemiological literature, reviewed in earlier sections, would inform the analysis of the risk of death from COVID-19, both for spatial areas and for specific social groups. We theorised that assets (reflecting both wealth and the overall size of the household, with joint household pooling larger assets such as vehicles) would act as a control variable. The asset index would control for wealth, which in turn is associated with getting good health care for each infected patient. At the same time, a low level of assets would act as a proxy for possible deficiencies in nutrients and economic resilience.

District-level variables include percentage of obesity and underweight. Since obese people are at risk of many other diseases, and underweight people are also widely at risk of vitamin deficiencies due to undernourishment, COVID-19 infections might result in high morbidity among them. Thus, higher percentage of obesity and underweight in a district (or a state) may result in higher mortality due to COVID-19.

We also have predictive objectives. We aimed to predict the death rate for each district given change over time in the rate of infection, age-standardised adjusted vulnerability to severe COVID-19 and multilevel analysis of migrant influx. We did not conduct a time-series forecasting but rather a cross-sectional, adjusted-for-confounders prediction. Future applications of the data-combining methods can create SEIR time-series predictions combined with various measures of social networks, normal social behaviour during the latent period, and aspects of transport and schooling, which might affect both contagion and the severity of disease. (For example, the migrants returning to village homes may have more severe cases of COVID-19.) We therefore fit a model to three data sources: NFHS, Census data at District level and COVID-19 death data. The maps in Fig. 5 illustrate the locations modelled, showing both the observed death rate and the predicted rates in districts of 11 states. In the 11 states analysed in detail here, the COVID-19 case count was cumulatively 205,000 (23 per 100,000 population), with 8358 deaths by June 17, i.e. 0.9 per 100,000 population (see details in Table A.3.). The population in the 11 states was 888.3 million compared with 1415 million across all of India. The cases covered in this paper (205,000) cover 2/3 of the cases in India in June (see Annex Table A.3; the total across India was 307,000 at that time in June 2020). The model with 11 states covered a wide range of both cities, mega-cities and villages.

One key reason for studying only part of India, not the whole, is our desire to publish maps; state and country boundaries are in some areas being politically contested, so it was most suitable to avoid certain boundaries. We examined the whole of the Gangetic plain and several surrounding states. These states have large cities containing most of the key SARS-CoV2 virus entry points. We omitted the Southern and North-eastern regions, where social norms, the social structure and cultural norms are rather distinctive. The cities of Bangalore and Chennai in South India are omitted from our model. The states of Kerala and West Bengal are not included, partly due to the historic differences in state level policy and politics. (Both had governments led by communist parties for over two decades.) The states of Gujarat, Madhya Pradesh and Maharashtra, with the highest case fatality ratios of 6%, 4% and 5%, respectively, were included in our model. Key cities of Mumbai, Delhi and Hyderabad were included. The overall case fatality ratio of our included states was 4% compared with 3% across all-India in June 2020 (see Online Annex Table A.3.). The proposed model can readily be extended to all states in India.

The specific datasets used here comprise two matrices. One contains a linked set of district data, one row per district, with NFHS and Census data concatenated. The second is the NFHS data at the individual level, with a district variable. In the NFHS data, we have one row per individual for women and a second row for their male spouses. (Typical analysis of the NFHS data focuses only on women, but we wanted men in the COVID-19 study.)

We use a Poisson regression with the outcome the COVID-19-related deaths in each of 345 districts for 11 states. The model aims to reveal potential risk factors related to COVID-19 mortality of individuals among women aged 15 to 49 and men aged 15 to 54.

To estimate model parameters and produce forecasts of mortality per district, we use Bayesian inference with weakly informative prior distributions. Bayesian inference permits estimating of complex hierarchical models. We use Hamiltonian Monte Carlo implemented in Rstan software, R interface to the program STAN for model analysis (Carpenter et al. 2017). Because of the large sample size, we used a generated total of 800 iterations with a burn-in sample of 300 iterations. The code for analysing the data is available at https://github.com/a-wis/Covid19-India.

There are two levels. Let *i* = 1, …, *N* denote individuals and *j* = 1,…,*G*, districts. Deaths in district *j* follow a Poisson distribution,

1 $$D_{j} \sim {\text{Poisson}}(\mu_{j} \cdot P_{j} ),$$

where *P*_*j*_ is an offset in form of a population in district *j* (in thousands), and *μ*_*j*_ is a death rate due to COVID-19, calculated as the average of the latent death risk variable (or vulnerability) over individuals *N*_*j*_ in district *j*:

2 $$\mu_{j} = {{\left[ {\sum\limits_{i = 1}^{{N_{j} }} {\mu_{ij} } } \right]} \mathord{\left/ {\vphantom {{\left[ {\sum\limits_{i = 1}^{{N_{3} }} {\mu_{ij} } } \right]} {N_{j} }}} \right. \kern-\nulldelimiterspace} {N_{j} }}.$$

We model the individual latent death risk with a two-level model:

3 $$\log \,\mu_{ij} = \alpha_{0} + \sum\limits_{k = 1}^{K} {\alpha_{k} X_{ijk} + \sum\limits_{l = 1}^{L} {\beta_{l} Z_{jl} } },$$

where *X*_*ij*_ = {female, urban, Scheduled Caste, Scheduled Tribes, smoking, ill-health, low assets} are individual-level predictors from the NFHS, and *Z*_*j*_ = {age 65+, migration, obesity, underweight} are district-level predictors.

To summarise this model, the dependent variable is the risk of death from COVID-19, and data on this variable take the form of counts of actual deaths (Eq. 1). The risk in turn is affected by two sets of factors: The *X* factors at the individual level and the *Z* factors at the district level. Equation 2 is a standard way to convert national Census population counts to death rates, or the risk rate. The hierarchical nature of the model allows linking both individual and district-level data to model a latent death rate, as well as using the variables from both levels as predictors.

The proportion of population aged 65+ and migration data (see previous section) is taken from India’s 2011 Census tables. The proportion who are obese or underweight are derived as the district mean of individual cases from married couples data in NFHS, as described earlier.

## Results

**Fig. 3:**
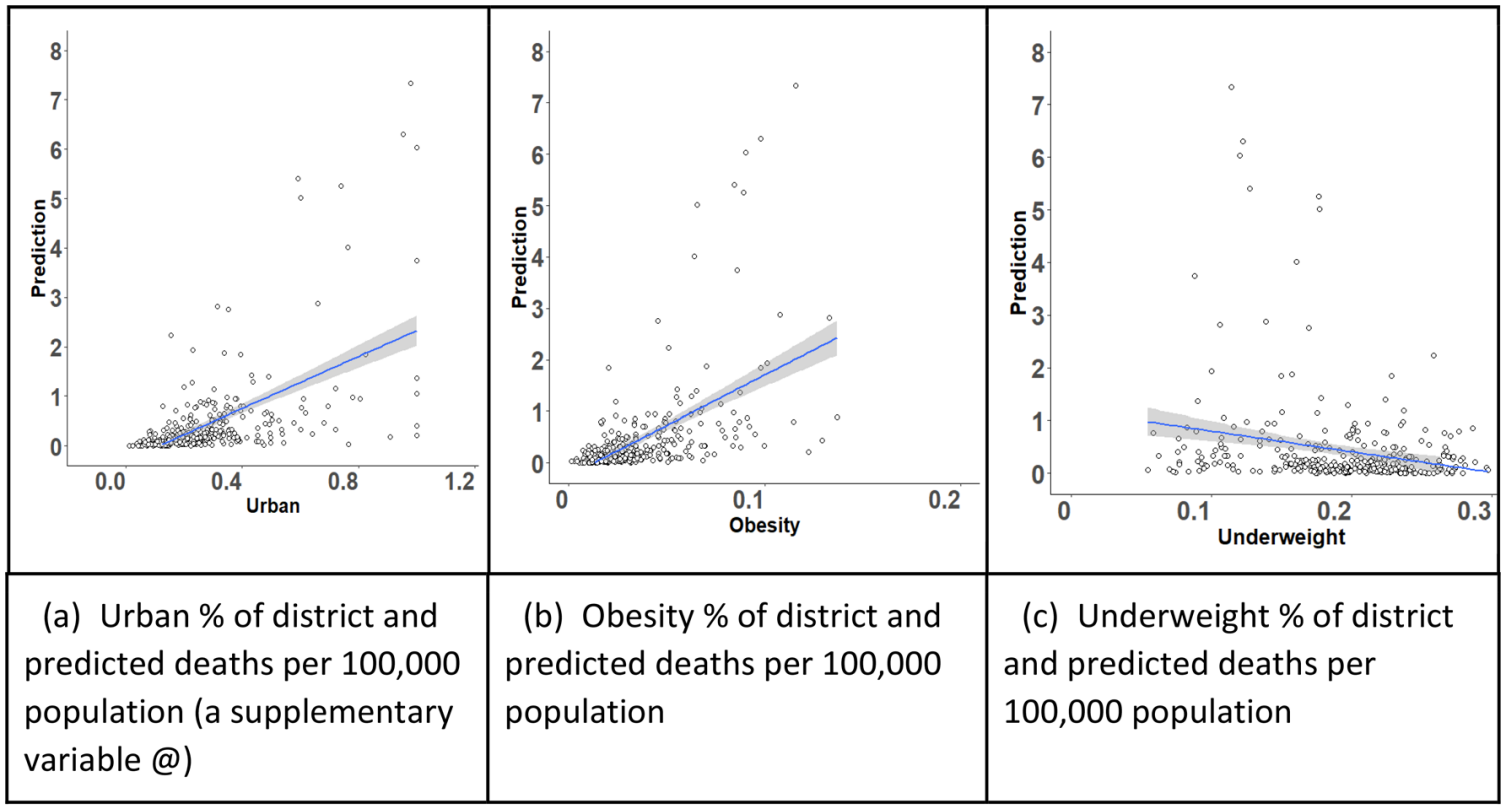
Predicted Indian deaths by district rate of urban residents, obesity and underweight. *Notes* Predictions use model 2. Prediction **a** uses the percent of district population who are urban residents (here scaled as ratio 0–1); **b** uses the percent of district population who are obese with BMI ≥ 30; **c** uses the percent of district population who are underweight with BMI < 18. Points are the predicted posterior means of each of 344 districts. Mumbai (predicted deaths 66) is excluded from these plots. Note that the smoothed curve here in each panel shows a linear trend line fit to the data in the plot, not the model results. @The urban % across a district is used in **a**, but individual urban residence was used in the actual regression models 1 and 2

**Fig. 4:**
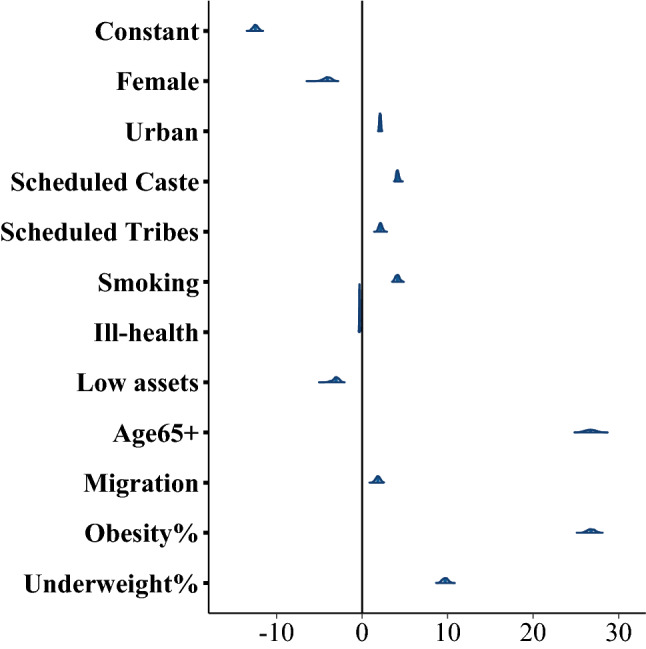
Posterior characteristics of Model 2 parameters. *Notes* The posterior means, posterior distributions and 95% Credible Intervals of each coefficient are graphed. See also Fig. A.1 in the Online Annex

The variables that cause potential risks of COVID-19 death are shown in Table 2. At the individual level, living in urban areas is a substantial risk factor. Its posterior mean coefficient is 2.88, with the 95% Credible Interval (CI: 2.69–3.11), based on Model 1. Figure 3a confirms that there is a strong correlation between predicted risks of death and the proportion of the urban populations. Up to June 2020, India’s COVID-19 spread and deaths have been concentrated in large urban areas (Fig. 4; Yadav and Bhattacharjee 2020). Social group differentials in the COVID-19 mortality rate are found after all other factors were controlled for, mean posterior slope *β* = 3.8 for SC people and *β* = 1.79 for ST people, i.e. the effect is more apparent among Scheduled Castes (SCs) than Schedules Tribes (STs) compared to all other groups. The reason for differences in COVID-19 effect in SCs and STs could arise due to differences in their location and economic status. The Scheduled Tribe people traditionally have had a very low out-migration rate (both rural to rural and rural to urban) and they are concentrated in geographically isolated areas with fairly low interaction outside their communities. The heightened risk of severe COVID-19 among marginalised social groups could be because of poor living conditions, sanitation, nutritional status and their long-term economic activities in some cases leading them to higher exposure to pollutants (Kim et al. 2020). Females have a negative coefficient, which could result from females having lower levels of outdoor travel and low economic participation outside homes.

**Table 2:** Regression result of the models for counts of COVID-19 deaths

Variables	Model 1 parameter estimates				Model 2 parameter estimates			
	mean	SD	2.5%	97.5%	mean	SD	2.5%	97.5%
Constant	− 12.87	0.27	− 13.45	− 12.36	− 12.46	0.29	− 13.01	− 11.91
**Individual-level variables**								
Female	− 3.89	0.45	− 4.79	− 3.1	− 4.05	0.45	− 4.94	− 3.24
Urban	**2.88**	**0.11**	**2.69**	**3.11**	**2.11**	**0.09**	**1.95**	**2.28**
SC (Dalit)	**3.8**	**0.13**	**3.56**	**4.09**	**4.16**	**0.16**	**3.89**	**4.49**
ST (Adivasi)	**1.79**	**0.18**	**1.46**	**2.14**	**2.16**	**0.2**	**1.78**	**2.58**
Smoking	**4.08**	**0.25**	**3.63**	**4.61**	**4.14**	**0.24**	**3.68**	**4.63**
Ill-health	− 0.26	0.03	− 0.33	− 0.2	− 0.29	0.04	− 0.36	− 0.22
LowAssets					− 3.08	0.4	− 4	− 2.42
**District-level variables**								
Age 65+*	**28.37**	**0.62**	**27.15**	**29.52**	**26.77**	**0.46**	**25.88**	**27.69**
Migration*	**2.23**	**0.26**	**1.72**	**2.77**	**1.85**	**0.27**	**1.29**	**2.38**
Obesity%	**26.77**	**0.48**	**25.82**	**27.67**	**26.68**	**0.64**	**25.41**	**27.93**
Underweight%	**9.49**	**0.36**	**8.8**	**10.21**	**9.71**	**0.34**	**9.07**	**10.37**

*Sources* Dependent variable (COVID-19 death) is from How India Lives (2020). Individual variables and obesity % and underweight % by district averaging are from NFHS 2015/16; and *age and migration data were obtained from Census data, 2011

*Notes* All the coefficients are significant. Rows in bold indicate increased risk of death. The dependent variable’s, *D*_*j*_, units are the counts of COVID-19 deaths. The population variable *P*_*j*_ acts as offset, leading to the predictions made in terms of rates *μ*_*j*_. Mean, SD, 2.5% and 97.5% refer to the mean, standard deviation and percentiles of the posterior distributions for the model parameters. The last four row variables are measured at district level, and the rest at individual level, reflecting the hierarchical model. Variables: At Level 1, Ill-health is an index of comorbidities. See Table 1 for details of SC, ST, smoker status and urban residence. At Level 2 (districts), migration indicates the proportion of in-migrants (0–9 years) living in the district. Age 65+ is the % (scaled 0 to 1) of population in each district who are over age 65. Obesity% is the percentage (scaled 0–1) of district population who are obese (BMI ≥ 30); and underweight% is the percentage (scaled 0–1) of district population who are underweight (BMI < 18). The reference category is coded “0” (as in Table 1). Model 1 vs 2: In Model 2 only, LowAssets indicates the lowest asset index quintile, coded as 1, with other quintiles set to 0 (see Table 1). This is an indicator of household poverty

The Model 2 results tend to validate the Model 1 slope estimates, because the addition of a key economic variable does not change the overall results very much. We also ran Model 1 and Model 2 estimations for August 2020 data which did not invalidate the results shown here. For a lack of space and a need for a more thorough analysis, we do not present those August 2020 results here.

Table 2 shows that some demographic variables are also closely associated with COVID-19-related mortality. The older-age group variable contributes to increased vulnerability. The mean of the population aged 65 and above is just 5% across 11 states, and so the coefficient of the variable, Age 65+ is large (posterior mean = 28.37, 95% CI = 27.15–29.52). According to the Periodic Labour Force Survey, or PLFS 2018/19 data (NSSO 2020), a larger proportion of older-age (65+) people live in rural areas (67% in rural vs 33% in urban areas), based fundamentally upon the predominantly rural population in India.

Furthermore, the percentage of migrants in the district is also positively related to the potential risks of death (mean *β* = 2.23, 95% CI = 1.72–2.77). Districts with a high percentage of in-migrants such as Mumbai, Ahmedabad and Delhi have more jobs and movement of people, and accordingly, their risk level increases. Particularly vulnerable are those living in slums and temporary shelters without adequate living space, water and sanitation facilities to maintain physical distance and livelihood security.

The regression includes many health-related variables, which are smoking, the ill-health index, obesity and being underweight. Smoking is one of the most influential health indicators that represent a high risk regarding COVID-19 mortality. Individuals who smoke are vulnerable to severe COVID-19 (mean *β* = 4.08, 95% CI = 3.63–4.61). COVID-19 is a respiratory virus, which could significantly affect people who have a weak lung function. Also, the obesity ratio in a district helps explain the increased death risk due to COVID-19. People who have BMI equal to 30 or more have higher vulnerability to severe COVID-19. The result shows that the coefficient of the variable "obesity%" is 26.77 (CI: 25.82–27.67). Obese people are at risk of many other diseases, and so COVID-19 infections might result in significant morbidity among them. Figure 3b shows that there is a slight positive correlation between the obesity rate of each district and the predicted number of deaths. In Fig. 3c, a smaller effect is also shown for the underweight rate of each district. Those districts with fewer underweight people have fewer deaths from COVID-19. Figure 3c is based on all factors combined, whereas in Table 2 the separate impact of the percentage underweight is that it increases the probability of a COVID-19 death. Thus, after controlling for other variables, the district’s underweight percentage has a positive impact on the number of deaths in the 11 states covered. Thus, lifestyle and health conditions strongly affect the outcome of having the COVID-19 disease in India.

These results show that the ‘pre-existing’ conditions should include obesity and being underweight. Another conclusion is that a Gini coefficient measure of inequality is likely to capture, and perhaps hide, the two forms of body-mass index (BMI) variation (high and low). Future data-combining research will have to carefully tease out the distinct BMI-related and economic factors that arise under high levels of inequality in India.

The percent of people underweight (BMI < 18) is found affecting the death rate upward. Although the direct covariance is weakly negative (Online Annex Table A.4), after controlling other variables, it turns out that the underweight percent is positively related to high risks of severe COVID-19. It has a positive posterior coefficient of 9.49 (95% CI: 8.8–10.2). People below the normative threshold of BMI = 18 are widely at risk of vitamin deficiencies or anaemia due to undernourishment, and thus are at risk of severe COVID-19 and even death. Note that in Models 1 and 2, the variables, "obesity%" and "underweight%" are measured at district level. Obesity and underweight at the individual level were tested in Models 3 and 4 (see Online Annex Table A.5) and no association was found. This may likely be due to the lack of data on the BMI of the people who died.

Individuals’ obesity or underweight condition were strong risk factors in severe COVID-19 cases as the other socio-economic and demographic variables, notably living in urban areas, and being a smoker. Smoking was defined inclusively here as any use of tobacco. The impact of obesity or being underweight also had a small effect compared with age and migration status. The ill-health index is negatively associated with the COVID-19 deaths, after controls, contradicting the correlation which was positive between the ill-health index and the death rate of each district. This implies that other factors have a stronger positive association, and they in turn are mediating a negative association of deaths with prior health conditions.

In Model 2, as a variant, we include the variable for wealth, which does not disrupt the findings of Model 1. Asset poverty was negatively associated with severe COVID-19, after all other controls. In brief, asset poverty is negatively correlated with urban residence, and the rural areas had not had as many deaths as urban areas, so the asset index was not explaining deaths well in the model.

Figure 4 shows the relative size of each effect, posterior distributions and the Credible Intervals (CI) (also in Table 2). Bayesian CIs are somewhat analogous to frequentist confidence intervals, and here they are very narrow due to the large sample sizes. The CIs correspond well to the actual variances observed. Online Annex Figure A.1. shows that in Model 2, which uses a variable controlling for assets, these means are not de-stabilised.

**Fig. 5:**
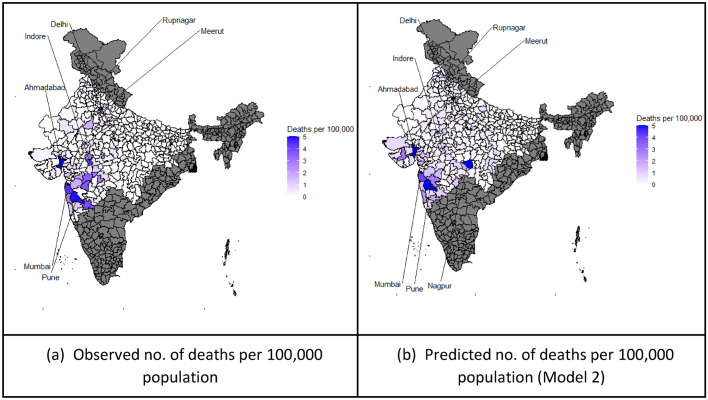
Map of India’s Observed and Predicted Number of Deaths. Notes: For greater coherence we modelled 11 states rather than all states, dark grey being parts not included; figures greater than 5 are rounded down to 5 here. *Source* https://www.howindialives.com/gram/coronadistricts/ (update date: June 17, 2020). *Map source*: projects.datameet.org/maps/districts, accessed Sept. 2020

In Fig. 5, we show the district-wise predicted outcome of COVID-19 deaths and the actual reported number of deaths for 11 states as of 17 June 2020. Higher risks of severe COVID-19 were found in Maharashtra, Gujarat and Delhi. They have high urban populations and more transportation links. The risks of COVID-19-related deaths are strongly related to the number of COVID-19-infected cases. Among the selected states, Mumbai has the highest risks of all for severe COVID-19. Its observed number of deaths reaches 110 per 100,000 population, yet the model predicts it as 66. Other districts with the highest predicted death rate within their respective states are South West of the National Capital Region, 8.96; Mumbai suburban in Maharashtra, 6.03; Ahmedabad in Gujarat, 5.26; Rupnagar in Punjab, 1.94; and Ghaziabad in Uttar Pradesh, 0.97.

It is a limitation of the model that transport links, as well as information on sanitation conditions and access to health care are not measured here. Further, there is no information about the key characteristics of those who died, such as age and sex. Finally, including interaction effects could improve the model fit.

## Discussion: World Health Organisation (WHO) versus Disaggregated Policy Advice

The WHO policy strategy offers a global common-denominator of advice about improved sanitation and hygiene; distancing and isolating/shielding; and avoiding this particular virus. The WHO aims to offer global advice, which appears at first glance nomothetic, so we give critical comments on three aspects. Our review of models had shown that nomothetic assumptions often assumed lawlike patterns across different nations, which might not be valid.

First, the smoking issue. The WHO (2020c) argues that smoking is associated with severe COVID-19 and thereby causes increased mortality among hospitalised COVID-19 patients. Our study also provides clear evidence of a positive relation. There is also a strong male bias in smoking in India. This male bias would be specific to India. This male bias might also need to be explored further as perhaps being a social-group and social-class-specific aspect. Passive smoking has also got to be considered as a health risk. Furthermore, pollution at the district or city level has not yet been put into the model (see also Kim et al. 2020). The pollution aspect was not part of the WHO's advice, yet it turns out to be relevant for the severe COVID-19. Thus more research is needed around this topic. For development experts, gender sensitivity is also needed in the policy response to both smoking and pollution issues.

Secondly, the WHO (2020d) also argues urban settings are riskier because of a high density of population and intense transport links. Informal settlements and social marginalisation are related concerns because COVID-19 transmission rates may be high as a result. More support should be given to marginal groups such as in-migrant workers in urban settings. We conclude that separate treatment is needed for managing the risk of infection versus the risk of severe COVID-19 cases and death. While linked, the pathways of cause are distinct (Fig. 2).

Thirdly, the issues around obesity and being underweight have had little discussion in the India case. The WHO advice has not gone into the precursors of either one. These weight issues are distinct, but both are related to social stigma. There is a risk of desirability bias which can cause them to be ignored. Being overweight places pressure on the heart and the circulation system. Those who are underweight may be at risk of other conditions, notably anaemia and calcium deficiency. Yet, there are also social-group differences to consider, so that normative conclusions are not easy to derive. Each government, whether Indian or state government, needs to address the weight and lifestyle issues specific to its residents.

At the policy level, to be prepared for potential risks of severe COVID-19, identifying the most vulnerable people, understanding their needs, hearing their authentic priorities, and providing adequate services through national and local collaborations are necessary. A focus on older-age groups seems less justified for India than looking at the diverse sources of risk themselves. Beyond the WHO advice on sanitation, policy targeting may also aim partly to encourage safe practices in households, firms and farms. Dis-aggregating information could help reduce the case fatality ratio.

## Conclusions

This paper innovates by demonstrating the feasibility of a method of combining data from random and non-random sources. We applied the method in the case of 11 states of India. The paper also reviewed the range of modelling options, which are being used to examine patterns of contagion and severity of COVID-19 cases.

When applying the proposed methods, a good theoretical foundation offers a solid grounding for choosing variables and testing hypotheses. Development theories range from trickle-down neoliberalism to structuralist democratic socialism, but the theoretical foundation of data combining is at a meta-level. In this case, our meta-theory involves making transdisciplinary assertions cutting across medical and health, economics and socio-cultural disciplines. This is common in three specific development theories: the idea of development as both policy and practice; ideas about development as the evolution of wellbeing (capabilities theory); and human development theory. The models in this paper tended to suggest that assets were not sufficient indices to predict severe COVID-19, but that assets needed to be seen within a broader model of vulnerability. We will comment on each of the three development theories in turn.

First, the idea of development as policy and practice sets up the agents of development as both individuals and corporate bodies. Development is not specific to the global ‘south’ but rather is a common method of improving lives in many countries. This approach coheres well with the WHO, who recommends that we learn from the regions of the world that had the epidemic earlier in the pandemic. For improved public health we would look at improvements in health delivery, review the structure of health services, examine how the society created vulnerabilities to the pandemic and help local policy makers to improve their area’s resilience to this epidemic. The models we create can be helpful to policy makers anywhere and they aim to inform evidence-based policy.

Second, the idea of development as wellbeing is usually presented as a long-term, multidimensional approach to human good lives, not just as conceptions but as lived experience. Here, development is inhibited by multidimensional poverty, and poverty enabled this virus to attack certain minority ethnic groups and slum dwellers, plus, in India’s case, the many in-migrants who do not live with extended families. The ‘wellbeing’ literature has a strong focus on measuring both subjective and objective aspects of wellbeing in synergy with each other. The new recession will cause further reductions in wellbeing. The capability approach usually breaks up the achievement of wellbeing into various domains: the health domain is one but housing, jobs, sanitation, being politically active i.e. participating and other domains are also important. This pandemic has shown that a failure of entitlements in a single domain can create barriers to all the others, and cause a rapid decline in wellbeing. In an epidemic, even the well-off people cannot always buy good health. In India, our model showed that being overweight, and obesity in particular, has been associated with more COVID-19 deaths, though it may not be a direct causal link. What is more, worse future patterns in rural areas could be observed, based on the association of COVID-19 vulnerability with being underweight. Underweight people are more prevalent in the rurally dominated states such as Uttar Pradesh and Madhya Pradesh (North-Central region), see Fig. 5. Thus, the link between the wellbeing theory and our model is a close one.

Thirdly, human development theory has been built upon the wellbeing argument to state that what constitutes development is the fuller emergence of actualised human beings. The main outcome focus in human development theory is multifaceted wellness, often proxied by the Human Development Index indicators (HDI). The HDI uses people’s achieved education, income per capita and longevity to gauge the aggregate level of development for a group or a region. During the COVID-19 era, all three of these are threatened directly. Human development is going downhill in India. Ameliorating policies are suggested from within all three tracks of the HDI index: getting children back to school or schooled from home; increasing earnings and creating opportunities for microenterprises; and directly improving health care and sanitation to raise longevity back upward. In India, several long-term trends of improvement in these key areas have been set back already by the disease. Our model showed that the areas with more people aged 65 and above were indeed the ones with higher mortality due to COVID-19 in the March-to-June 2020 stages. Furthermore, after controlling for that, age itself appeared irrelevant but a range of lifestyle and medical conditions increased or decreased a person’s vulnerability to severe COVID-19.

These results might seem anodyne yet they are evidence-based, and the results vary considerably by place and by social group. The pandemic has shown the strengths of the three transdisciplinary approaches to development, and the weaknesses of economic theories centred on commercial behaviour only (e.g. GDP per capita, trade flows and foreign investment). It may be worth noting also that the development research in an interdisciplinary setting has strengths that can improve health care outcomes all over the world.

The data-combining method can be applied in other developing-country contexts e.g. Pakistan, Sri Lanka, Bangladesh. Variations on the method include an "L-shaped" option (a new random survey using a subset of the original national survey, but with additional variables); the Delphi method for eliciting expert information that could be used to inform prior distributions for the model parameters; and other sources for well-informed priors as opposed to flat (weakly informative) priors. We discuss each briefly. First, we can construct an L-shaped dataset, using fresh random quantitative data on death counts, or using phone-survey data. Non-random data would be less useful; administrative data are highly useful. From this matrix, we predict and improve the estimates of the impact of COVID-19 using a larger, national survey. Second, we can use a multi-round ‘expert questionnaire’ known as the Delphi method to gather information from experts for the evidence base. This information can be used to construct prior distributions for parameters in a statistical model. Thirdly, we could also use well-informed priors based on other analyses. In the COVID-19 debate, much of such analysis has rested upon purely China data (e.g. based on Wuhan data from Feb–March 2020), or China and Italy, or the Diamond Princess cruise ship data. We argued that these outside sources must be used carefully, and not simply via the lawlike nomothetic assumption that each Indian district would have the same social structure or the same relationship of severe-COVID-19 to the age structure of exposure. It is possible to use external data to inform initial analyses. Then, relevant, preferably random-sample-based data specific to the country of interest need to be used to make further investigations, possibly with prior distributions informed by the findings from other countries' data. The best data need to be disaggregated, and Census results for 2021 that enable online use, user-driven crosstabulations, and perhaps even a record-level secure analysis of random samples of cases (see e.g. Williamson et al. 2020) would be potentially useful in future for India and other countries where administrative data sources are not typically released to the public.

## Electronic supplementary material

Below is the link to the electronic supplementary material.

Electronic supplementary material 1 (DOCX 68 kb)

## Footnotes

1 *ibid.* URL https://ourworldindata.org/coronavirus-data-explorer?zoomToSelection=true&deathsMetric=true&totalFreq=true&perCapita=true&smoothing=0&country=~IND&pickerMetric=location&pickerSort=asc, accessed 14/7/2020.

2 https://www.who.int/data/gho/data/themes/theme-details/GHO/body-mass-index-(bmi)
