# Supplementary material for: Hierarchical Modelling of COVID-19 Death Risk in India in the Early Phase of the Pandemic
Source: Eur J Dev Res. 2020 Dec 15;32(5):1476–503. doi: 10.1057/s41287-020-00333-5 (PMC7737421; doi:10.1057/s41287-020-00333-5)
Supplement: Supplementary file 1 — Electronic supplementary material 1 (DOCX 68 kb) [file 41287_2020_333_MOESM1_ESM.docx]

Special Issue EJDR on Covid-19 (2020)

Hierarchical Modelling of COVID-19 Death Risk in India in the Early Phase of the Pandemic

Online Annex

**Table A.1**. Sampling Procedure of the Indian National Family Health Survey 2015/16

| The NFHS 2015/16 uses a two-stage stratified cluster sample – one for cluster selection and the other for households, so the sampling weights are the product of the two stages selection probabilities. Cluster selection is based on the type of residency (urban-rural) and SC/ST population using the 2011 Census information. Then 22 households are selected in each cluster with an equal probability systematic selection (IIPS and ICF, 2017, pp.609–611). The number of interviewed women (aged 15–49) is much larger than the number of men in the whole dataset. Because of the disproportionate composition of male-female samples, males and females are given different sampling weights within clusters and households. The NFHS males and females sampling weights are based on the 2011 Census population, so we need to use the population growth rate between 2011 and 2020 to adjust the weight level to the 2020 population. Adjusted sampling weights are divided by the mean weight and rounded up to the nearest integer greater than 0. Calculated integer weights range between 1 and 28, representing the relative weights of each sample. |
| --- |

**Table A.2.** Correlation Matrix

|  | Cases_Pop  /1000 | Deaths_  Pop/1000 | Female | Urban | SC | ST | Smoking | Ill-health | Low  assets | Obesity | Under-  weight | Age65+ | Migration | Obesity  % | Underweight  % |
| --- | --- | --- | --- | --- | --- | --- | --- | --- | --- | --- | --- | --- | --- | --- | --- |
| Cases_Pop/1000 | 1.00 |  |  |  |  |  |  |  |  |  |  |  |  |  |  |
| Deaths_Pop/1000 | 0.82 | 1.00 |  |  |  |  |  |  |  |  |  |  |  |  |  |
| Female | 0.00 | 0.00 | 1.00 |  |  |  |  |  |  |  |  |  |  |  |  |
| Urban | 0.09 | 0.10 | 0.00 | 1.00 |  |  |  |  |  |  |  |  |  |  |  |
| SC | 0.02 | 0.02 | 0.00 | -0.05 | 1.00 |  |  |  |  |  |  |  |  |  |  |
| ST | -0.03 | -0.03 | 0.00 | -0.17 | -0.20 | 1.00 |  |  |  |  |  |  |  |  |  |
| Smoking | -0.02 | -0.02 | -0.54 | -0.07 | 0.02 | 0.08 | 1.00 |  |  |  |  |  |  |  |  |
| Ill-health | 0.01 | 0.02 | 0.02 | 0.06 | -0.01 | -0.05 | -0.03 | 1.00 |  |  |  |  |  |  |  |
| Low assets | -0.06 | -0.06 | 0.00 | -0.30 | 0.05 | 0.30 | 0.13 | -0.06 | 1.00 |  |  |  |  |  |  |
| Obesity | 0.02 | 0.02 | 0.05 | 0.12 | -0.03 | -0.06 | -0.07 | 0.10 | -0.09 | 1.00 |  |  |  |  |  |
| Underweight | -0.01 | -0.02 | 0.06 | -0.11 | 0.02 | 0.09 | 0.03 | -0.04 | 0.14 | -0.08 | 1.00 |  |  |  |  |
| Age65+ | 0.02 | 0.00 | 0.00 | 0.00 | 0.01 | -0.02 | -0.01 | -0.01 | -0.13 | 0.02 | -0.04 | 1.00 |  |  |  |
| Migration | 0.13 | 0.22 | 0.00 | 0.19 | -0.03 | 0.03 | -0.06 | 0.03 | -0.25 | 0.06 | -0.06 | 0.30 | 1.00 |  |  |
| Obesity% | 0.19 | 0.16 | 0.00 | 0.25 | 0.06 | -0.22 | -0.11 | 0.06 | -0.31 | 0.13 | -0.09 | 0.13 | 0.50 | 1.00 |  |
| Underweight% | -0.07 | -0.11 | 0.00 | -0.19 | -0.10 | 0.26 | 0.10 | -0.07 | 0.24 | -0.07 | 0.15 | -0.21 | -0.30 | -0.58 | 1.00 |

Notes: cases_pop/1000= COVID-19 cases per 1000 population, deaths_pop/1000= COVID-19 related deaths per 1000 population

**Table A.3.** Descriptive Information For 11 States

| States | Deaths | Deaths per 1,000 population | Cases | Cases per 1,000 population | Case fatality rates | 2020 Population / 1,000 |
| --- | --- | --- | --- | --- | --- | --- |
| Bihar | 43 | 0.000 | 6,829 | 0.05 | 0.01 | 128,447 |
| Chhattisgarh | 6 | 0.000 | 1,261 | 0.04 | 0.00 | 30,962 |
| Delhi | 10 | 0.000 | 3,824 | 0.19 | 0.00 | 20,177 |
| Gujarat | 1,514 | 0.021 | 24,124 | 0.34 | 0.06 | 71,476 |
| Haryana | 118 | 0.004 | 8,253 | 0.27 | 0.01 | 30,195 |
| Jharkhand | 9 | 0.000 | 3,284 | 0.08 | 0.00 | 39,865 |
| Madhya Pradesh | 473 | 0.005 | 10,735 | 0.12 | 0.04 | 86,328 |
| Maharashtra | 5,432 | 0.042 | 113,031 | 0.87 | 0.05 | 129,815 |
| Punjab | 67 | 0.002 | 3,186 | 0.10 | 0.02 | 31,328 |
| Rajasthan | 280 | 0.003 | 16,799 | 0.20 | 0.02 | 82,180 |
| Uttar | 406 | 0.002 | 13,361 | 0.06 | 0.03 | 237,540 |
| **Total (11 states)** | **8,358** | **0.009** | **204,687** | **0.23** | **0.04** | **888,312** |
| **Total (All-India)** | **9,856** | **0.007** | **307,451** | **0.22** | **0.03** | **1,415,646** |

Notes: deaths or cases = the number of deaths or cases due to COVID-19 at district (17th of June 2020); case fatality rates= the number of COVID-19 deaths/ the number of COVID-19 cases; 2020 population was estimated using the monthly growth rate of the population in each district between the last censuses of 2001 and 2011.

**Figure A.1** Posterior characteristics of parameters from Model 1 and Model 2

| 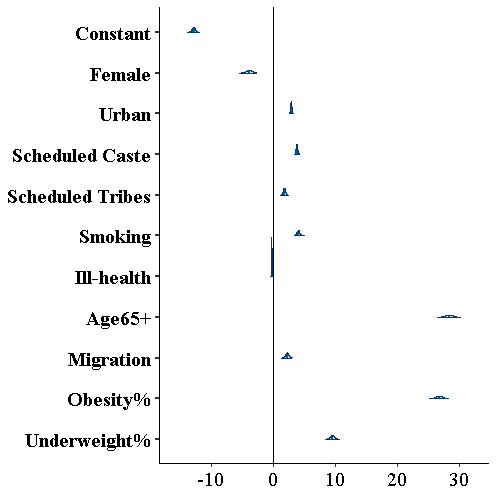 | 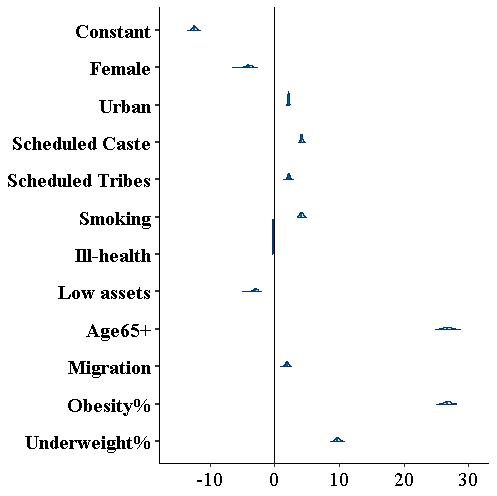 |
| --- | --- |
| Model 1 | Model 2 |

Notes: The posterior mean, whole predictive distribution and 95% credible intervals of each coefficient is graphed; the others are the same as in Table 3. Based on own calculations using *rstan* package in R.

**Table A.4**. Covariance matrix of the variables used in the models

|  | death_j | female | urban | sc | st | smoking | ill-health | low assets | obesity | underweight | age65+ | migration | Obesity% | Underweight% |
| --- | --- | --- | --- | --- | --- | --- | --- | --- | --- | --- | --- | --- | --- | --- |
| death_j | 11830.89 |  |  |  |  |  |  |  |  |  |  |  |  |  |
| female | 0.11 | 0.25 |  |  |  |  |  |  |  |  |  |  |  |  |
| urban | 5.35 | 0.00 | 0.21 |  |  |  |  |  |  |  |  |  |  |  |
| sc | 0.18 | 0.00 | -0.01 | 0.16 |  |  |  |  |  |  |  |  |  |  |
| st | -1.31 | 0.00 | -0.03 | -0.03 | 0.12 |  |  |  |  |  |  |  |  |  |
| smoking | -0.97 | -0.13 | -0.02 | 0.00 | 0.01 | 0.23 |  |  |  |  |  |  |  |  |
| ill-health | 1.24 | 0.01 | 0.03 | 0.00 | -0.02 | -0.02 | 1.49 |  |  |  |  |  |  |  |
| low assets | -3.12 | 0.00 | -0.06 | 0.01 | 0.04 | 0.03 | -0.03 | 0.18 |  |  |  |  |  |  |
| obesity | 0.49 | 0.00 | 0.01 | 0.00 | 0.00 | -0.01 | 0.02 | -0.01 | 0.04 |  |  |  |  |  |
| underweight | -0.46 | 0.01 | -0.02 | 0.00 | 0.01 | 0.01 | -0.02 | 0.02 | -0.01 | 0.12 |  |  |  |  |
| age65+ (district) | -0.01 | 0.00 | 0.00 | 0.00 | 0.00 | 0.00 | 0.00 | 0.00 | 0.00 | 0.00 | 0.00 |  |  |  |
| Migration (district) | 0.68 | 0.00 | 0.00 | 0.00 | 0.00 | 0.00 | 0.00 | 0.00 | 0.00 | 0.00 | 0.00 | 0.00 |  |  |
| obesity% (district) | 0.59 | 0.00 | 0.00 | 0.00 | 0.00 | 0.00 | 0.00 | 0.00 | 0.00 | 0.00 | 0.00 | 0.00 | 0.00 |  |
| Underweight% (district) | -0.56 | 0.00 | -0.01 | 0.00 | 0.01 | 0.00 | 0.00 | 0.01 | 0.00 | 0.00 | 0.00 | 0.00 | 0.00 | 0.00 |

**Table A.5.** Regression Result for Indian Deaths: Models Including Individual Obesity and Underweight

|  | Model 3 | | | | | Model 4 | | | | |
| --- | --- | --- | --- | --- | --- | --- | --- | --- | --- | --- |
| Variables | mean | median | sd | 2.50% | 97.50% | mean | median | sd | 2.50% | 97.50% |
| Constant | -12.67 | -12.67 | 0.24 | -13.18 | -12.2 | -11.92 | -11.92 | 0.23 | -12.39 | -11.47 |
| **Individual-level variables** | | |  |  |  |  |  |  |  |  |
| Female | -4.41 | -4.38 | 0.42 | -5.27 | -3.66 | -4.41 | -4.38 | 0.46 | -5.36 | -3.57 |
| **Urban** | **4.63** | **4.63** | **0.17** | **4.33** | **4.97** | **3.94** | **3.94** | **0.15** | **3.67** | **4.26** |
| **SC** | **2.86** | **2.86** | **0.07** | **2.73** | **3** | **2.88** | **2.88** | **0.07** | **2.76** | **3.01** |
| **ST** | **0.54** | **0.54** | **0.14** | **0.27** | **0.79** | **0.63** | **0.64** | **0.14** | **0.33** | **0.88** |
| **Smoking** | **3.73** | **3.71** | **0.22** | **3.32** | **4.2** | **3.72** | **3.72** | **0.21** | **3.34** | **4.13** |
| Ill-health | -0.57 | -0.56 | 0.08 | -0.74 | -0.44 | -0.61 | -0.6 | 0.09 | -0.82 | -0.47 |
| LowAssets |  |  |  |  |  | -3.62 | -3.61 | 0.48 | -4.62 | -2.73 |
| Obesity | -3.25 | -3.22 | 0.5 | -4.3 | -2.36 | -3.17 | -3.16 | 0.49 | -4.19 | -2.28 |
| Underweight | -2.28 | -2.23 | 0.43 | -3.22 | -1.57 | -2.89 | -2.85 | 0.5 | -4 | -2 |
| **District-level variables** | | |  |  |  |  |  |  |  |  |
| **Age65+** | **33.19** | **33.21** | **0.58** | **32** | **34.28** | **32.01** | **31.99** | **0.58** | **30.89** | **33.16** |
| **Migration** | **4.73** | **4.73** | **0.22** | **4.31** | **5.17** | **4.02** | **4.02** | **0.23** | **3.57** | **4.47** |

Notes: bold letters indicate risk of death (with positive figures); models with individual obesity/underweight

**Table A.6.** The Highest 20 COVID-19-Death Districts (June 17, 2020)

A.6.(a) Top 20 Districts, Ranked by the **Predicted** Number of COVID-19 Deaths (Model Estimates)

Note: Deaths per 100,000; 11 states. For convenience, predictions based on Model 2 are provided. The 2.5^th^ and 97.5^th^ percentiles of the predictive distribution for *µ_j_* define the 95% Predictive Intervals.

| No | State | District | Observed | **Predicted**  **(mean)** | 2.5^th^ percentile | 97.5^th^ percentile |
| --- | --- | --- | --- | --- | --- | --- |
| 1 | Maharashtra | Mumbai | 110.35 | 65.54 | 62.96 | 68.04 |
| 2 | Delhi | South West | 0.03 | 8.96 | 8.55 | 9.43 |
| 3 | Delhi | East | 0.00 | 8.94 | 8.43 | 9.48 |
| 4 | Delhi | North | 0.00 | 7.34 | 6.98 | 7.72 |
| 5 | Delhi | North West | 0.02 | 6.30 | 6.00 | 6.62 |
| 6 | Maharashtra | Mumbai Suburban | 0.01 | 6.03 | 5.77 | 6.30 |
| 7 | Maharashtra | Pune | 4.88 | 5.40 | 5.16 | 5.66 |
| 8 | Gujarat | Ahmadabad | 13.96 | 5.26 | 5.06 | 5.47 |
| 9 | Maharashtra | Nagpur | 0.23 | 5.01 | 4.86 | 5.17 |
| 10 | Maharashtra | Thane | 4.36 | 4.02 | 3.75 | 4.32 |
| 11 | Delhi | West | 0.00 | 3.75 | 3.56 | 3.95 |
| 12 | Gujarat | Rajkot | 0.11 | 2.88 | 2.76 | 3.01 |
| 13 | Punjab | Rupnagar | 0.12 | 2.81 | 2.66 | 2.98 |
| 14 | Maharashtra | Ahmadnagar | 0.24 | 2.76 | 2.61 | 2.90 |
| 15 | Maharashtra | Ratnagiri | 1.17 | 2.24 | 1.94 | 2.57 |
| 16 | Punjab | Shahid Bhagat Singh Nagar | 0.16 | 1.94 | 1.82 | 2.08 |
| 17 | Maharashtra | Sangli | 0.36 | 1.88 | 1.79 | 1.97 |
| 18 | Gujarat | Surat | 1.29 | 1.84 | 1.67 | 2.01 |
| 19 | Maharashtra | Wardha | 0.07 | 1.84 | 1.69 | 1.99 |
| 20 | Maharashtra | Aurangabad | 3.62 | 1.42 | 1.37 | 1.47 |

A.6.(b) Top 20 Districts, Ranked by the **Observed** Number of COVID-19 Deaths

Note: Deaths per 100,000; 11 states. For convenience, predictions based on Model 2 are provided.

| No | State | District | **Observed** | Predicted  (mean) | Lower (0.025) | Upper  (0.975) |
| --- | --- | --- | --- | --- | --- | --- |
| 1 | Maharashtra | Mumbai | 110.35 | 65.54 | 62.96 | 68.04 |
| 2 | Gujarat | Ahmadabad | 13.96 | 5.26 | 5.06 | 5.47 |
| 3 | Maharashtra | Pune | 4.88 | 5.4 | 5.16 | 5.66 |
| 4 | Maharashtra | Thane | 4.36 | 4.02 | 3.75 | 4.32 |
| 5 | Madhya Pradesh | Indore | 4.15 | 1.16 | 1.12 | 1.21 |
| 6 | Maharashtra | Solapur | 3.83 | 0.95 | 0.9 | 1 |
| 7 | Maharashtra | Aurangabad | 3.62 | 1.42 | 1.37 | 1.47 |
| 8 | Maharashtra | Jalgaon | 3.51 | 0.64 | 0.6 | 0.68 |
| 9 | Madhya Pradesh | Burhanpur | 2.88 | 0.17 | 0.16 | 0.18 |
| 10 | Madhya Pradesh | Ujjain | 2.85 | 0.68 | 0.65 | 0.71 |
| 11 | Maharashtra | Akola | 2.8 | 0.44 | 0.41 | 0.47 |
| 12 | Maharashtra | Raigarh | 2.77 | 1.14 | 1.08 | 1.22 |
| 13 | Madhya Pradesh | Bhopal | 2.41 | 0.95 | 0.91 | 0.99 |
| 14 | Haryana | Gurugram | 2.2 | 0.04 | 0.03 | 0.05 |
| 15 | Maharashtra | Dhule | 2.1 | 0.32 | 0.31 | 0.34 |
| 16 | Maharashtra | Nashik | 1.79 | 1.29 | 1.11 | 1.48 |
| 17 | Rajasthan | Jaipur | 1.66 | 0.53 | 0.51 | 0.56 |
| 18 | Uttar Pradesh | Meerut | 1.53 | 0.8 | 0.77 | 0.84 |
| 19 | Haryana | Faridabad | 1.52 | 0.19 | 0.18 | 0.21 |
| 20 | Gujarat | Gandhinagar | 1.45 | 0.36 | 0.34 | 0.39 |

**Table A.7.** Rstan Code for the Model

Available on URL <http://github.com/a-wis/Covid19-India>
